# Reliability of Obstacle-Crossing Parameters during Overground Walking in Young Adults

**DOI:** 10.3390/s24113387

**Published:** 2024-05-24

**Authors:** Matthias Chardon, Fabio Augusto Barbieri, Pascal Petit, Nicolas Vuillerme

**Affiliations:** 1 AGEIS (Autonomie, Gérontologie, E-Santé, Imagerie et Société), Université Grenoble Alpes, 38000 Grenoble, France; matthias.chardon@gmail.com (M.C.); pascal.petit@univ-grenoble-alpes.fr (P.P.); 2 Human Movement Research Laboratory (MOVI-LAB), Department of Physical Education, Sao Paulo State University (UNESP), Bauru 17033-360, SP, Brazil; 3 Institut Universitaire de France, 75005 Paris, France

**Keywords:** reliability, obstacle-crossing, gait

## Abstract

We aimed to evaluate the intra-session relative and absolute reliability of obstacle-crossing parameters during overground walking in young adults, and to determine the number of trials required to ensure reliable assessment. We analysed data from 43 young male adults who were instructed to walk at a self-selected velocity on a pathway and to step over an obstacle (height = 15 cm; width = 80 cm, thickness = 2 cm) three times. Spatial–temporal gait parameters of the approaching and crossing phases (i.e., before and after the obstacle) and obstacle clearance parameters (i.e., vertical and horizontal distance between the foot and the obstacle during crossing) were computed using a three-dimensional motion analysis system. Intraclass correlation coefficients were used to compute the relative reliability, while standard error of measurement and minimal detectable change were used to assess the absolute reliability for all possible combinations between trials. Results showed that most spatial–temporal gait parameters and obstacle clearance parameters are reliable using the average of three trials. However, the mean of the second and third trials ensures the best relative and absolute reliabilities of most obstacle-crossing parameters. Further works are needed to generalize these results in more realistic conditions and in other populations.

## 1. Introduction

Reliability does represent a crucial consideration as a methodology for application in gait analysis. Indeed, if gait analysis is intended to serve as an outcome measure for diagnostic, monitoring, or therapeutic purposes, the level of reliability for each computed gait parameter has to be established.

This is certainly one of the reasons that has prompted scientists and researchers to focus on the reliability assessment of gait measurements in both healthy [1,2,3,4,5] and pathological [3,6,7,8,9,10,11] populations. In previous studies conducted on overground/flat conditions in healthy patients [2] and stroke individuals [6,7], speed and stride length [2,6,7], as well as cadence and gait cycle time [7], showed good reliability. In addition, knee, hip, and ankle angles showed good to excellent reliability in healthy individuals [1] and adults with spinal cord injury [6].

However, few studies have investigated the number of trials needed to ensure reliable intra-session gait assessment [2,9,12]. For instance, Soulard et al. (2021) [2] showed that three trials were enough to ensure reliable gait assessment during the 10 m walk test in both single and dual-task conditions in healthy adults [2]. Interestingly, the common feature of the above-mentioned gait reliability studies is the assessment of unobstructed level walking tasks. However, not much is known about the reliability of gait parameters under more challenging conditions, such as crossing physical obstacles while overground walking (obstructed walking task). This observation is somewhat surprising since obstacle-crossing performance during walking has been widely studied using vision/camera-based systems in various populations, including children [13,14], older healthy adults [15,16], individuals with obesity [17], and people with Parkinson’s disease [18,19,20,21], multiple sclerosis [22], traumatic brain injuries [23], or stroke [24,25]. This observation is also of great concern since obstacles are likely encountered during community ambulation in an everyday environment (e.g., obstacles on sidewalks or in corridors of railway or subway stations [26]). In addition, it is widely agreed that measuring clearance parameters during the performance of the obstacle-crossing task is of significant clinical importance, assuming that lower toe clearances [27] and horizontal distances [28] could result in a high probability of foot contact and hence an increased risk of stumbles, trips, and falls.

To our knowledge, no study has yet investigated the reliability of these widely used gait and clearance parameters during obstacle-crossing in young healthy adults, whereas the reliability of gait parameters computed using vision/camera-based systems has previously been assessed during unobstructed walking in healthy adults (e.g., [29,30,31,32]). To address some of the aforementioned limitations, the purpose of the present work was to evaluate the reliability, relative and absolute, of obstacle-crossing parameters during overground walking in young adults. We further aimed to determine the number of trials required to ensure reliable obstacle-crossing measurements. Indeed, determining the number of trials required to ensure reliable measurement is a prerequisite to allow comparisons between groups at a single time point [33]. Note that this question has been the subjects of a number of studies in a variety of applications fields, including human movement, sport, and health, to name a few (e.g., [2,34,35,36,37,38,39,40,41,42,43,44,45,46,47]). In particular, intra-session reliability can favourably be used to assess the number of trials to ensure reliable measurements [2,9,38,48] to optimize gait assessments in clinical practice.

## 2. Materials and Methods

This study is a secondary analysis of previously published obstacle-crossing studies [22,49,50].

### 2.1. Participants

Data from 43 Brazilian males (age = 29.1 ± 5.91 years old, body mass = 76.3 ± 9.97 kg, body height = 1.77 ± 0.06 cm, BMI = 24.4 ± 2.72 kg/m^2^, mean ± SD) were analysed. Participants had no diagnosis of muscular or neurodegenerative disease. Participants were informed about the experimental procedures and signed informed consent. The study was approved by the University Institution Review Board (authorization number: CAAE#99191318.0.0000.5398). This study and all methods were performed in accordance with the Declaration of Helsinki.

### 2.2. Experimental Protocol

Participants were instructed to walk barefoot at a self-selected velocity on an 8.5 m long pathway and to step over a physical obstacle (height = 15 cm; width = 80 cm, thickness = 2 cm) placed in the middle of the pathway. To ensure that participants crossed the obstacle with the right leg as the leading limb, the starting point of each trial was adjusted, and at least two strides were completed before crossing the obstacle. Participants completed three trials.

### 2.3. Gait Assessment

Kinematic data were recorded using ten infrared cameras (Vicon Motion System^®^, Oxford, UK, 200 Hz). Two markers were positioned at the top of the obstacle. Also, four markers were placed on the lateral aspect of the calcaneus and head of the second metatarsus of the right limb and the medial aspect of the calcaneus and head of the second metatarsus of the left limb. Conventional labelling of the limbs was used: the leading foot crossed the obstacle first, and the trailing foot crossed second. Signals were filtered with a low-pass (6 Hz) Butterworth filter of fifth-order (zero-lag). These pre-processed signals were used to compute the following parameters:

Spatial–temporal gait parameters (length, width, duration, velocity, and double-support time) of the approaching phase (stride before the obstacle) and of the crossing phase (step over the obstacle);

Leading and trailing foot placement prior to the obstacle (horizontal distance from the second metatarsal marker to the obstacle);

Leading and trailing vertical toe foot clearance during obstacle-crossing (vertical distance at the moment that the second metatarsal marker was above the obstacle);

Leading and trailing foot placement after the obstacle (horizontal distance from the heel marker to the obstacle).

### 2.4. Statistical Analysis

To examine the potential difference between trials for each obstacle-crossing parameter, a repeated measures analysis of variance (RM-ANOVA) was first carried out. In cases where a difference was observed (type I error rate set at α = 0.05), a pairwise comparison post hoc test was performed to compare differences between pairs of trials, using the Benjamini–Hochberg approach to account for multiple testing.

To assess relative reliability across the three trials, the intraclass correlation coefficient (ICC) based on a two-way random effects model (absolute agreement, ICC(2.1)) was calculated. ICC(2.1) values inferior to 0 were considered ‘poor’, between 0.01 and 0.20 as ‘slight’, between 0.21–0.40 as ‘fair’, between 0.41–0.60 as ‘moderate’, between 0.61–0.80 as ‘substantial’, and 0.80–1.00 as ‘almost perfect’ relative reliability [51].

To assess absolute reliability across the three trials, the standard error of measurement (SEM) and the minimum detectable change (MDC) were computed. SEM, with the same unit as obstacle-crossing parameters, corresponds to the absolute measure of the variability of the errors of measurements and informs on the precision of obstacle-crossing parameters of individual participants [52]. SEM was calculated using the following formula [53]: SEM = SD√(1-ICC(2.1)), where SD is the standard deviation of the obstacle-crossing parameters from all participants and ICC(2.1) is the relative reliability. MDC is the minimum value for which a difference can be considered “real”. MDC was calculated using the following formula [54]: MDC = SEM × 1.96 × √2. The SEM% and MDC% were also expressed as a percentage of the mean for each obstacle-crossing parameter. Lower values for SEM% and MDC% indicate higher absolute reliability. Precisely, SEM% values were considered ‘low’ (SEM% ≤ 10%) or ‘high’ (SEM% > 10%) [55], while MDC% values were considered ‘low’ (MDC% ≤ 20%), ‘acceptable’ (20% < MDC% < 40%), or ‘high’ (MDC% ≥ 40%) [56].

To quantify the agreement between pairs of trials for each of the 16 obstacle-crossing parameters, Lin’s concordance correlation coefficient (CCC) and associated 95% confidence intervals [57,58], as well as the Shieh exact test for agreement [59], were used. The CCC indicates how close the measurement pairs fall to the 45-degree line (perfect agreement). Hence, the CCC is a measure of the reproducibility of measurements. The CCC ranges from −1 to 1, with perfect agreement at 1 and perfect discordance at −1. Agreement was considered ‘poor’ for CCC values < 0.40, ‘moderate’ for CCC values ranging from 0.40 to 0.70, and ‘good’ for CCC values > 0.70 [60].

Limits of agreement (LOA) and Bland–Altman plots of the differences between trials and their arithmetic mean were used to assess the magnitude of disagreement between trials for each obstacle-crossing parameter. LOA allows the quantification of bias and the determination of a range of agreement, within which 95% of the differences between one measurement and the other are included. The bias is significant when the line of equality is not within the 95% CI of the mean difference. The smallest worthwhile change (SWC) was used to determine the maximum allowed difference between trials presented in Bland–Altman plots [61]. The SWC provides a method to evaluate a real change in performance between trials. If the SWC is low and included in the LOA or Shieh 95% CI, it indicates a low variation between trials. Two trials are considered in agreement if the LOA or Shieh exact test does not exceed the maximum allowed difference between trials (SWC).

All statistical analyses were performed using R software 4.3.1^®^ (R Core Team, Vienna, Austria) for Windows 10©. The list of all R packages used is provided in the Supplementary Materials.

## 3. Results

### 3.1. Difference between Trials

Most obstacle-crossing parameters exhibited no significant differences between trials. The only three exceptions were observed for the stride width and the double support time (*p* = 0.04, eta-squared = 0.01) during the approaching phase, and for the trailing foot horizontal distance after the obstacle (*p* = 0.02, eta-squared = 0.02). For these three parameters, pairwise *t*-tests showed that trial 1 (T1) differed from trials 2 and 3. Stride width was lower in T1 compared to T2 and T3 (*p* = 0.02 for T1–T2, *p* = 0.04 for T1–T3), double support time was greater for T1 compared to T2 and T3 (*p* = 0.05 for T1–T2, *p* = 0.03 for T1–T3), while for the trailing foot horizontal distance after obstacle, T3 was higher compared to T1 and T2 (*p* = 0.03).

Table 1 provides the arithmetic mean and standard deviation of obstacle-crossing parameters for each trial and the mean of the three trials.

### 3.2. Absolute and Relative Reliabilities

**Figure 1:**
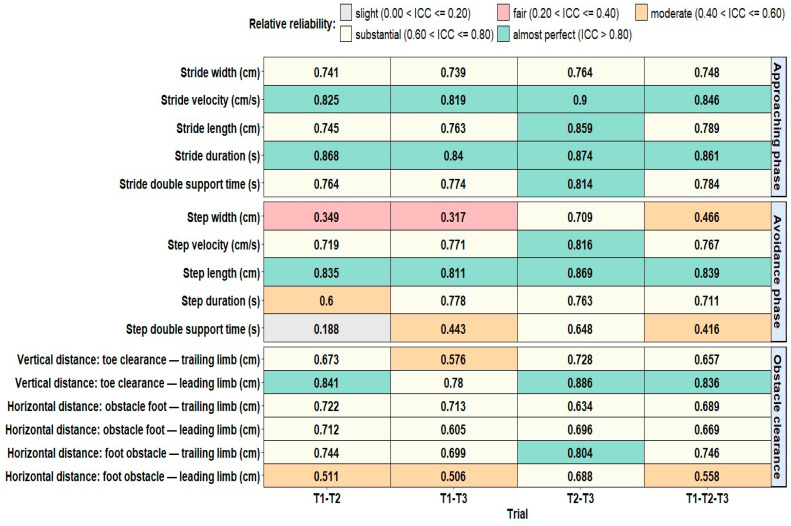
Mean ICC(2.1) for the means of trial 1–2, 1–3, 2–3 and 1–2–3 calculated for each obstacle-crossing parameter.

Figure 1 and Table 2 present the ICC(2.1) values for each obstacle-crossing parameter for all comparisons of trials. Table 2 also provides the SEM, SEM%, MDC, and MDC%.

Overall, when pooling the values of the three trials, the relative reliability ranged from moderate to practically perfect, whereas the absolute reliability was good for nine out of the sixteen parameters. Furthermore, the highest relative and absolute reliabilities were found when pooling values from T2–T3, followed by T1–T2–T3, T1–T3, and T1–T2.

#### 3.2.1. Spatial–Temporal Gait Parameters during the Approaching Phase

Relative reliability: Regardless of the comparison of trials considered, spatial–temporal gait parameters during the approaching phase showed either substantial or almost perfect reliability. Indeed, ICC(2.1) ranged from 0.61 to 0.80 for stride width for all trial comparisons, for stride length, and double support time for T1–T2, T1–T3, and T1–T2–T3, while ICC(2.1) ranged from 0.81 to 1.00 for stride duration and stride velocity for all trial comparisons, and for stride length and double support time for T2–T3.

Absolute reliability: Regardless of the comparison of trials considered, SEM% and MDC% values were low for most of the spatial–temporal gait parameters during the approaching phase (SEM% < 10 and MDC% < 20%), with the exception of the stride width (SEM% > 10% and 20% < MDC% < 40%, for all trial comparisons), and MDC% of the double support time (MDC% = 20.4%, for T1–T2).

Number of trials to ensure reliable measurements: The comparisons of means of ICC(2.1) between trials showed that, for all spatial–temporal gait parameters during the approaching phase, slightly higher ICC(2.1) and lower SEM% and MDC% were obtained when pooling the second and third trials, compared to the first and second trials, the first and third trials, or the three trials.

#### 3.2.2. Spatial–Temporal Gait Parameters during the Crossing Phase

Relative reliability: Regardless of the comparison of trials considered, step length, step duration, and step velocity showed either substantial or almost perfect relative reliability. Indeed, ICC(2.1) ranged from 0.61 to 0.80, for step duration for all trial comparisons and for step velocity for T1–T2, T1–T3, and T1–T2–T3, while ICC(2.1) ranged from 0.81 to 1.00, for step length for all trial comparisons and for step velocity for T2–T3. Furthermore, step width and double support time showed slight relative reliability (ICC(2.1) = 0.19 for double support time for T1–T2), fair relative reliability (ICC(2.1) ranged from 0.21 to 0.40, for step width for T1–T2 and T1–T3), moderate relative reliability (ICC(2.1) ranged from 0.41 to 0.60 for step width for T1–T2–T3, and for double support time for T1–T3 and T1–T2–T3), or substantial relative reliability (ICC(2.1) ranged from 0.61 to 0.80 for step width and double support time for T2–T3 only).

Absolute reliability: Regardless of the comparison of trials considered, SEM% and MDC% were low or acceptable (SEM% < 10% and MDC% < 40%) for step length, duration, and velocity. Step width and double support time showed high SEM% and MDC% values (SEM% > 10% and MDC% > 40%), except for double support time when pooling the second and third trials with an acceptable MDC% (MDC% = 34.4%).

Number of trials to ensure reliable measurements: The comparisons of means of ICC(2.1) between trials showed that, for almost all spatial–temporal gait parameters during the crossing phase, slightly higher ICC(2.1) (except for step duration, the best relative reliability was found for T1–T3, ICC(2.1) = 0.78) and lower SEM% (all parameters) and MDC% (all parameters) were obtained when pooling the second and third trials, compared to the first and second trials, the first and third trials, or the three trials.

#### 3.2.3. Obstacle Clearance Parameters

Relative reliability: Regardless of the comparison of trials considered, obstacle clearance parameters showed either moderate, substantial, or almost perfect mean relative reliability. The highest ICC(2.1) values were found for leading toe clearance with substantial to almost perfect reliability (0.78 ≤ ICC(2.1) ≤ 0.89 for all trial comparisons), followed by trailing foot horizontal distance prior to the obstacle, and leading and trailing foot horizontal distance after the obstacle with substantial reliability (0.61 ≤ ICC(2.1) ≤ 0.80 for all trial comparisons). The lowest ICC values were found for the trailing foot toe clearance (moderate reliability, ICC(2.1) = 0.58, for T1–T3) and for the leading foot horizontal distance prior to an obstacle (moderate reliability, 0.51 ≤ ICC(2.1) ≤ 0.56, for T1–T2, T1–T3, and T1–T2–T3).

Absolute reliability: Regardless of the comparison of trials considered, SEM% and MDC% were low or acceptable for leading foot horizontal distance prior to the obstacle and trailing foot horizontal distance after the obstacle (SEM% < 10% and MDC% < 40%). Regardless of the comparison of trials considered, high SEM% values were found for leading and trailing foot toe clearance, leading foot horizontal distance after the obstacle, and trailing foot horizontal distance prior to the obstacle (for T1–T2, T1–T3, and T1–T2–T3). High MDC% (MDC% > 40%) values were observed for the leading (all trial comparisons) and trailing (T1–T2, T1–T3) limb toe clearances, as well as for the horizontal distance after the obstacle for the leading limb (T1–T3, T2–T3, T1–T2–T3).

Number of trials to ensure reliable measurements. The comparisons of means of ICC(2.1) between trials showed that, for almost all clearance parameters, slightly higher ICC(2.1) and lower SEM% and MDC% were obtained when pooling the second and third trials, compared to the first and second trials, the first and the third trials, or the three trials.

### 3.3. Agreement between Trials

When considering the maximum allowed difference between trials (SWC), regardless of the obstacle-crossing parameter, all pairs of trials were considered in agreement because the SWC was always within the LOA and Shieh ranges.

#### 3.3.1. Spatial–Temporal Gait Parameters during the Approaching Phase

Good agreement (CCC > 0.70) between each pair of trials was found for all spatial–temporal gait parameters during the approaching phase.

The best results were found for T2–T3 for all parameters, followed by T1–T2 and T1–T3 for stride width, stride duration, and stride velocity, or followed by T1–T3 and T1–T2 for stride length and double support time.

#### 3.3.2. Spatial–Temporal Gait Parameters during the Crossing Phase

Moderate (0.40 < CCC < 0.70) and good (CCC > 0.70) agreements between each pair of trials were found for all spatial–temporal gait parameters during the crossing phase, with the exception of the step width for T1–T2 (CCC = 0.34) and T1–T3 (CCC = 0.31), and of the double support time for T1–T2 (CCC = 0.19) with poor agreement between trials.

The best results were found when pooling values from T2–T3 for step length, step width, step velocity, and double support time (except for step duration, agreements between trials were better for T1–T3, CCC = 0.77 vs. CCC = 0.76 for T2–T3), followed by T1–T2 and T1–T3 for step length and step width, or followed by T1–T3 and T1–T2 for step velocity and double support time.

#### 3.3.3. Obstacle Clearance Parameters

Moderate (0.40 < CCC < 0.70) and good (CCC > 0.70) agreements between each pair of trials were found for all obstacle clearance parameters.

The best results were found when pooling values from T2–T3 for leading and trailing foot toe clearance and foot horizontal distance prior to the obstacle. However, for leading and trailing foot horizontal distance after obstacle, agreements between trials were better for T1–T2 (CCC = 0.71 vs. CCC = 0.69 for T2–T3 for leading horizontal distance after obstacle, and CCC = 0.72 vs. CCC = 0.63 for T2–T3 for trailing foot distance after obstacle), followed by T1–T2 and T1–T3.

**Figure 2:**
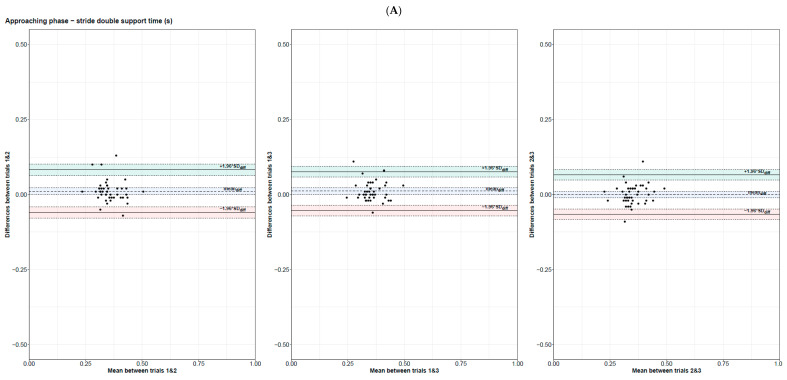

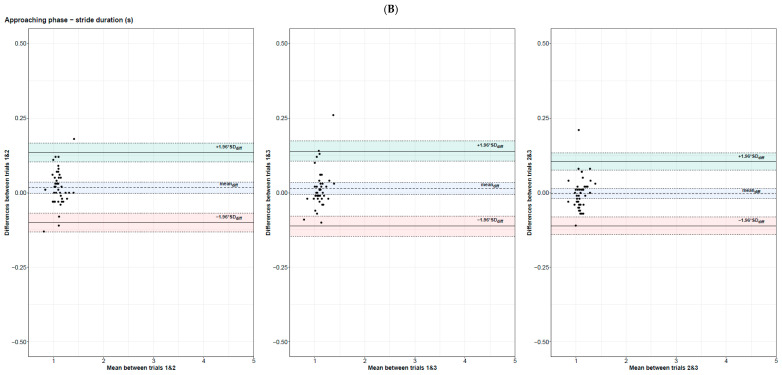

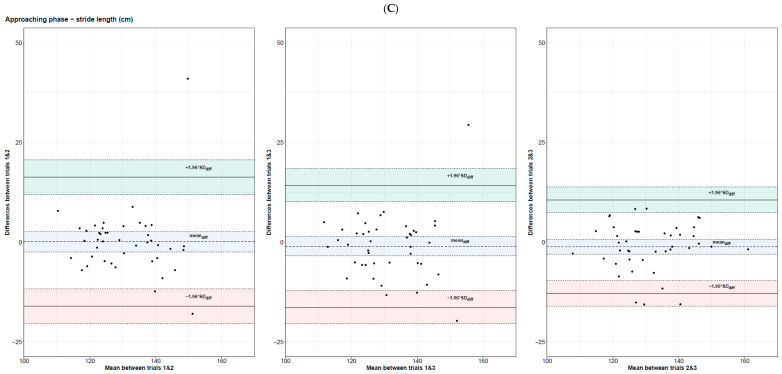

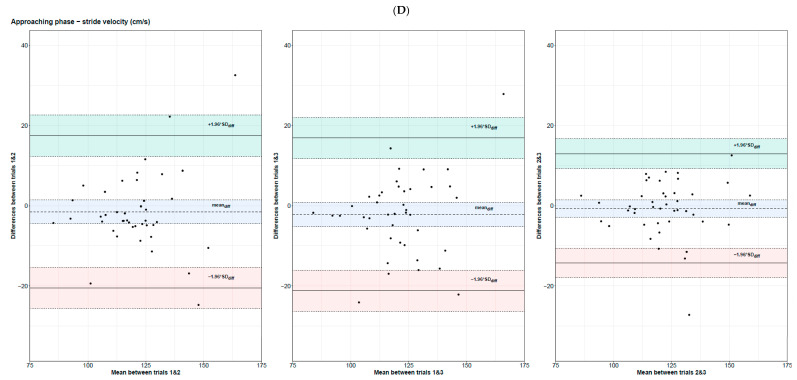

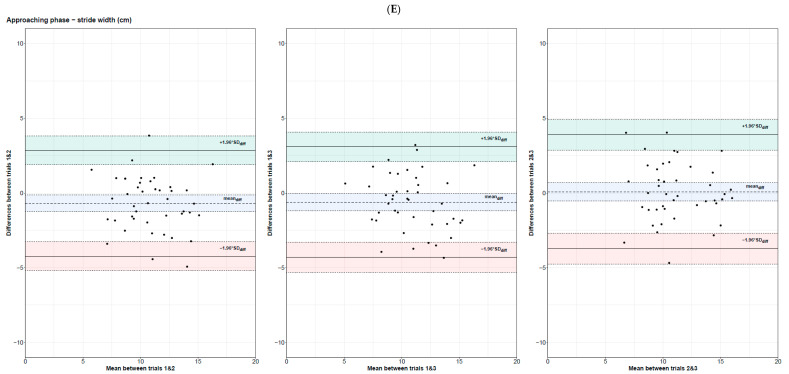

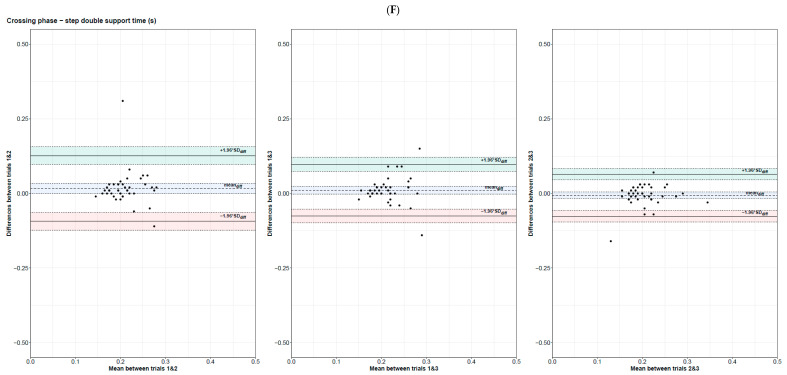

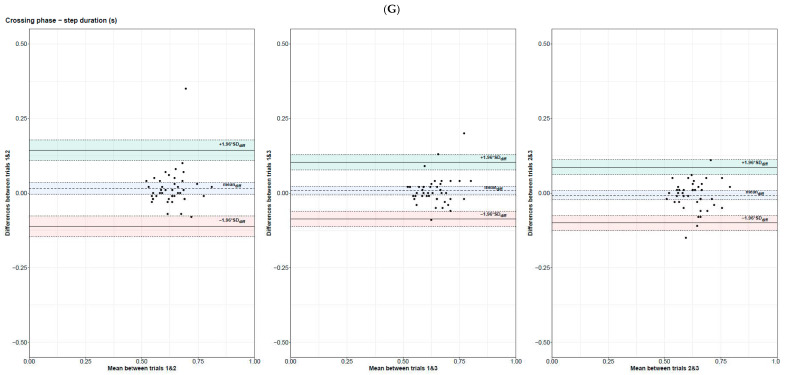

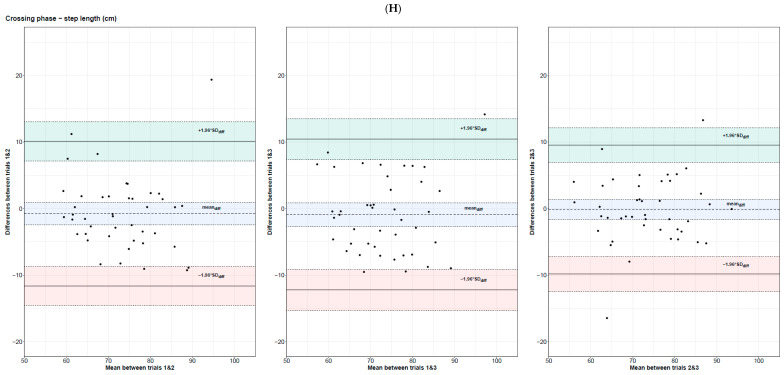

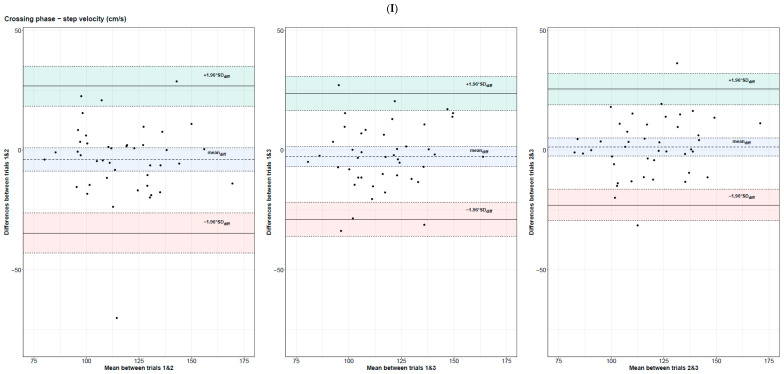

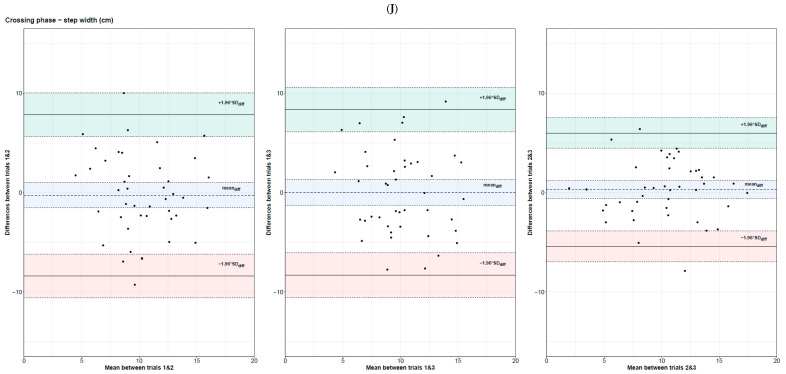

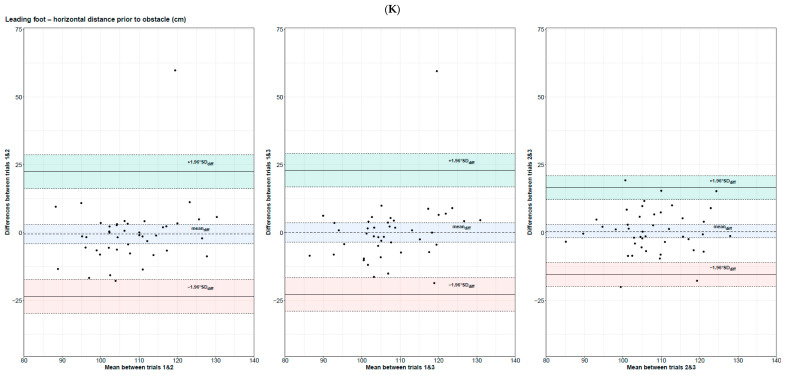

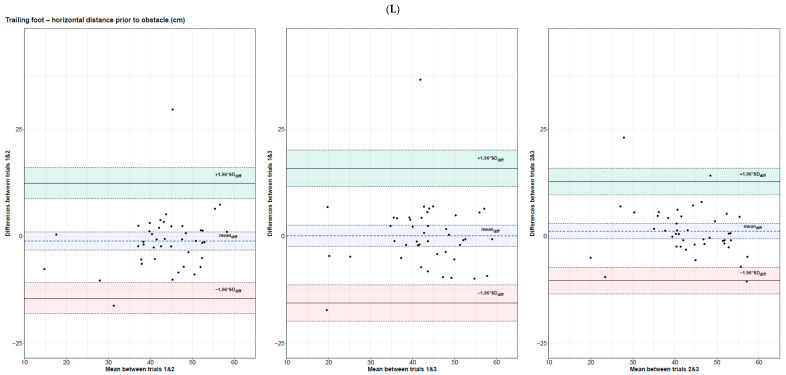

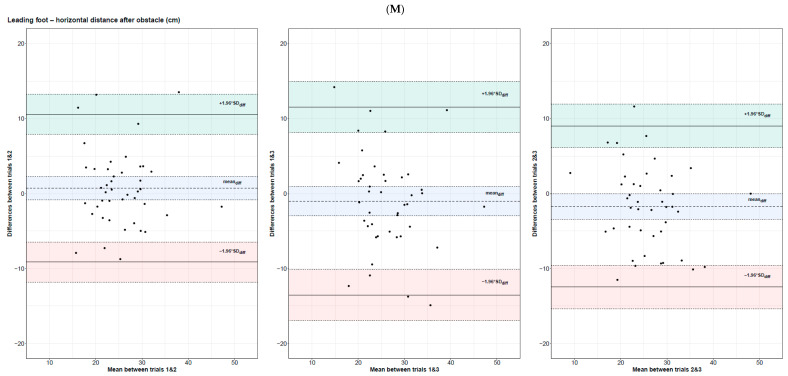

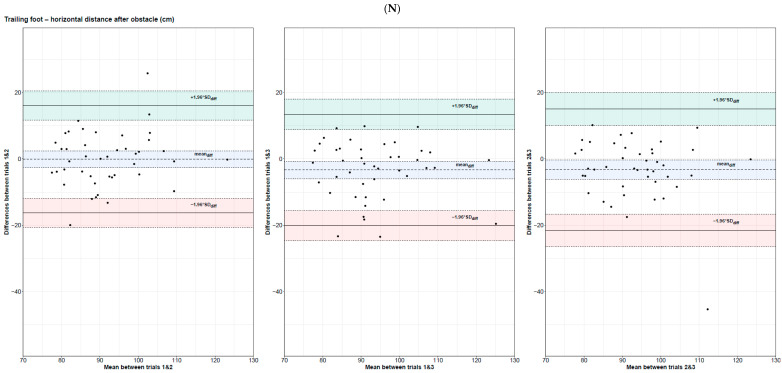

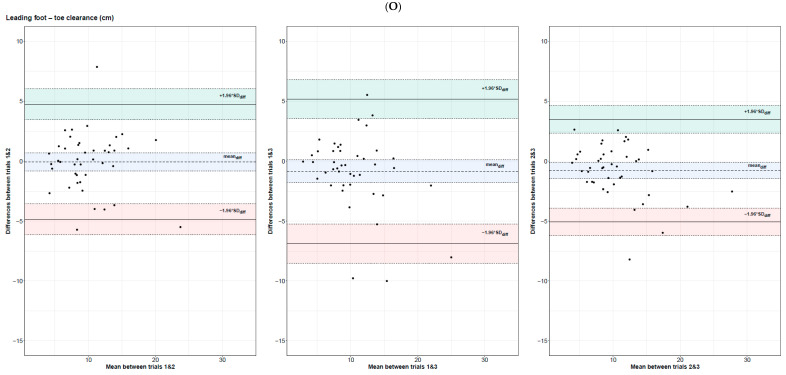

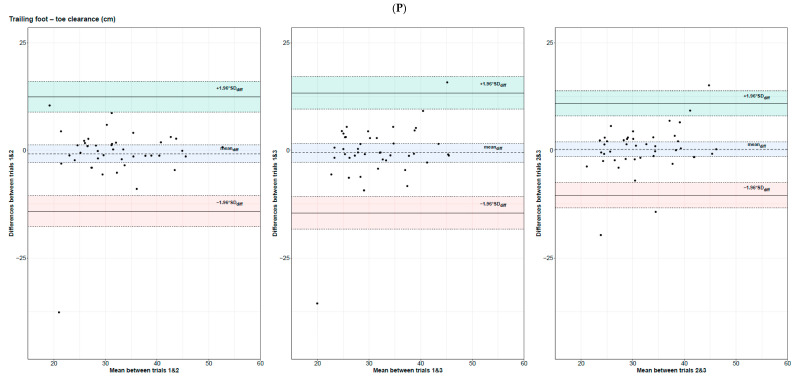
Bland–Altman plots for trial 1 and 2, 1 and 3, and 2 and 3 for gait parameters during the approaching phase (**A**–**E**), the crossing phase (**F**–**J**), and obstacle clearance parameters (**K**–**P**).

Table 3 presents the CCC, LOA, Shieh exact test agreement, and SWC. Figure 2 presents the Bland–Altman of each gait and obstacle-crossing parameter.

## 4. Discussion

The aim of this study was to evaluate the intra-session relative and absolute reliability of obstacle-crossing parameters during overground walking in young adults and to determine the number of trials required to ensure reliable assessment.

Overall, our results showed for the first time that spatial–temporal gait parameters and clearance parameters were reliable, except for the step width (between T1–T2 and T1–T3) and double support time (between T1–T2) during the crossing phase. In addition, for most spatial–temporal gait and clearance parameters, the reliability and agreement were better when pooling trials 2 and 3 than when the first trial was used for comparison. This suggests that when conducting an obstacle-crossing task in young adults—under the specific experimental conditions used in the present study, at least—a first practice trial should be performed before the experimental trials to enhance the reliability of the parameters studied, or that the first experimental trial should be discarded from data analysis.

### 4.1. Intra-Session Reliability during Obstacle-Crossing

#### 4.1.1. Approaching Phase

Relative reliability for all spatial–temporal gait parameters measured in young adults during the approaching phase was always either substantial or almost perfect. Regarding absolute reliability, all parameters except step width (SEM% ≥ 10%) exhibited good absolute reliability in all trial comparisons, and MDC% was low or acceptable for all gait parameters in all trial comparisons. Comparisons between trials showed that participants had a higher step width in the last two trials compared to the first one. Perhaps after the first trial, the strategy chosen was to increase their pre-crossing base of support after the trial to ensure more stability before the crossing phase. Regarding agreements between trials, results were considered good for all spatial–temporal gait parameters in all trial comparisons. Hence, young male adults had almost identical gait patterns during all trials during the approaching phase. In addition, for all spatial–temporal gait parameters, relative and absolute reliabilities and agreement between trials were better when using the mean of the second and third trials, compared to the first and second trials, to the first and third trials, or to the three trials. This could suggest that participants adapted a more stable/consistent gait pattern after the execution of the first trial, which could have been used as a learning/warming-up trial. These results are similar to those recently reported in healthy adults during unobstructed level walking [2]. In that study, 20 healthy participants performed three trials of a 10-m walking test, and spatial–temporal gait parameters were computed using inertial motor units (Physilog® 5, 200Hz GaitUp, Lausanne, Switzerland). Results showed that speed, double support, and stride length had almost perfect relative reliability (0.81 < ICC(2.1)). In addition, absolute reliability was good, with low SEM% (1.74% ≤ SEM% ≤ 6.58% in all trial comparisons) and low MDC% (4.82% ≤ MDC% ≤ 18.25% for all trial comparisons) for these three spatial–temporal gait parameters [2].

#### 4.1.2. Crossing Phase

Contrary to what was observed for the approaching phase, not all spatial–temporal gait parameters were found to have substantial or almost perfect relative reliability, good absolute reliability, or good agreement between trials.

The relative reliability of step length, duration, and velocity was substantial to almost perfect regardless of the trial comparisons considered. Conversely, step width and double support time showed slight to moderate relative reliability (0.19 ≤ ICC(2.1) ≤ 0.47), with the worst results when the first trial was included. Regarding absolute reliability, both step width and double support time showed poor reliability in all trial comparisons, except the MDC% of double support time, which was acceptable for the T2–T3 comparison (MDC% = 34.4). In addition, agreements between trials were poor for step width (T1–T2 and T1–T3) and double support time (T1–T2), compared to the other spatial–temporal parameters (moderate or good) between each pair of trials. Comparison between trials showed no differences in step width or double support time during the crossing phase (*p* < 0.05). One hypothesis is that the lower reliability found for step width and double support time parameters during the crossing phase compared to the approaching phase could be explained by the more unusual nature of this step compared to the approaching phase (i.e., walking on flat ground) and due to the higher demand of the task in neuromuscular [62] and cognitive resources [63] than flat ground walking. Also, medial lateral dynamic balance during the crossing phase might be harder to maintain, so it is difficult for the participants to maintain a stable behaviour for crossing step width. However, it is worth noting that agreements between trials were better for all spatial–temporal gait parameters, with the exception of step duration (for which the best value was obtained for T1–T3) when pooling values from the second and third trials.

#### 4.1.3. Obstacle Clearance

Relative reliability for all clearance parameters measured in young adults was either moderate to almost perfect (0.51 ≤ ICC(2.1) ≤ 0.89), and the reliability was enhanced from substantial to almost perfect when pooling the second and third trials (0.63 ≤ ICC(2.1) ≤ 0.89).

However, the absolute reliability was low for four clearance parameters (leading and trailing foot horizontal distance after obstacle, trailing foot horizontal distance after obstacle, and leading foot toe clearance) for T1–T2, T1–T3, and T1–T2–T3, and three clearance parameters for T2–T3 (leading and trailing foot toe clearance and leading foot horizontal distance after obstacle). In addition, MDC% was high (i.e., 40% ≤ MDC%) for two clearance parameters for T1–T2 (leading and trailing foot clearance), T2–T3 (leading foot toe clearance and horizontal distance after obstacle), and T1–T2–T3 (leading foot toe clearance and horizontal distance after obstacle), and for three clearance parameters for T1–T3 (leading and trailing foot toe clearance and leading foot horizontal distance after obstacle).

On the one hand, in general, clearance parameters were less reliable than spatial–temporal gait parameters measured during the approaching and crossing phases (with the exception of stride width and double support time during the crossing phase). As mentioned, the more unusual nature of this step compared to flat ground walking might have played a role in individuals’ behaviour and clearance strategy. In addition, it is possible that the higher cognitive load and precise motor control during obstacle-crossing compared to unobstructed walking [63] may contribute to the reduced reliability of clearance parameters. On the other hand, similar to what was observed for spatial–temporal gait parameters computed before and after the obstacle, for most obstacle clearance parameters, both relative and absolute reliabilities were better when using the mean of the second and third trials, compared to the first and second trials, to the first and third trials, or to the three trials. This reinforces the idea of a learning/warming-up effect after the first trial for individuals when they perform an obstacle-crossing task. However, comparisons between trials showed that trailing foot horizontal distance after the obstacle was higher in the third trial compared to the first and second. Together, these results suggest that, contrary to spatial–temporal parameters, individuals might need more than three trials to have reliable behaviour. Regarding agreements between trials, moderate to good agreement was found for all parameters between each pair of trials. Similarly to relative and absolute reliabilities, the best results were found for four out of six clearance parameters (leading and trailing toe clearance and distance prior to obstacle) when pooling the second and third trials. However, agreements between trials were better for T1–T2 for both leading and trailing foot distances after the obstacle.

As shown by measures of agreement (CCC and Bland–Altman, see Figure 2), there should not be any systematic bias in measurements since no obvious relation existed between the difference and the mean of any pair of two trials. In addition, the LOA were relatively small, with few to no outliers depending on the obstacle clearance parameter considered.

Having high absolute reliability (10% < SEM%) indicates that there were intra-individual variabilities between trials.

### 4.2. Study Limitations, Strengths, and Perspectives

To the authors’ best knowledge, this is the first study that assessed the reliability, both relative and absolute, and agreements between trials of spatial–temporal gait parameters and obstacle clearance parameters in young adults. It should be noted that although previous studies have reported either the absolute or relative reliability of these parameters (e.g., [64,65,66]), none of them addressed our specific research questions. Indeed, we aimed to evaluate the intra-session relative and absolute reliability of obstacle-crossing parameters during overground walking in young healthy adults, and to determine the number of trials required to ensure reliable assessment. Contrary to us, the study from Punt et al. (2017) assessed virtual obstacle-crossing task during treadmill walking in stroke survivors [64], while Grinberg et al. (2021) did not assess obstacle-crossing parameters during overground walking [66], and Said et al. (2009) did not involve young healthy adults [65]. In addition, the sample size of the present study is relatively high (n = 43), compared to previously conducted gait reliability studies [1,2,3,5,8,11,38]. At this point, it is important to mention that, fully in line with the scientific roadmap we have set up to assess the intra-session reliability of the 10 m walk test [2], we focused on young healthy adults as a first step to provide normative reference values for a young healthy population (e.g., see [67,68,69]). Naturally, the reliability must now be further tested in other populations with different socio-demographic, anthropometric, clinical, and lifestyle characteristics, including individuals who are overweight or obese (e.g., see [70] for a review), children, adolescents, middle age, and the elderly (e.g., see [71] for a review), and pathological populations (e.g., see [72,73,74] for reviews) who may also present different levels of fatigue (e.g., see [75] for a review) or physical fitness (e.g., see [76] for a review).

Finally, the present findings highlighted that most spatial–temporal and obstacle clearance parameters were reliable across the three trials and that better relative and absolute reliabilities, as well as agreements between trials, were found when pooling the second and third trials. Nevertheless, several limitations warrant further consideration and caution regarding the interpretation of the results.

The reliability and agreement were assessed during a single experimental session, which prevented us from studying the inter-session reliability. In addition, only young healthy males aged between 20–40 years old were included, and only one experimental condition (15 cm height obstacle; self-selected walking speed; single-task condition) was assessed. In other words, strictly speaking, our conclusion applies only to the population tested and the experimental conditions used in the present study. These experimental conditions could be considered as relatively mild and easy in that the task consisted of crossing a physical obstacle 15 centimetres high at a comfortable speed without any other particular constraint. Further works are thus needed to generalize them in more realistic conditions. For instance, it could be interesting to replicate this reliability study while walking over different and challenging obstacle paradigms that may predispose the participants to an increased risk of foot contact with the obstacle [77,78,79] and/or with individuals with balance and gait disorders for whom the consequences of imbalance and tripping over obstacles could be more dramatic [80,81]. Hence, these specific issues deserve investigations, which are included in our immediate plans. Another potential future investigation would be to perform more than three trials of obstacle-crossing. This would probably enhance the reliability of all parameters, especially the clearances with the obstacle. This might enable us to determine the minimum number of trials to be carried out, and the number of trials from which data analysis should begin in order to obtain reliable and valid data.

## 5. Conclusions

Most spatial–temporal gait parameters and obstacle clearance parameters computed using a three-dimensional motion analysis system in young adults during an obstacle-crossing task are reliable using the average of three trials. Our findings further suggest using the mean of the second and third trials to ensure the best relative and absolute reliabilities of most obstacle-crossing parameters. However, our findings only apply to the population tested and to the relatively mild and easy experimental conditions used in the present study. Further works are thus needed to generalize them in more realistic conditions and in other populations.

## Figures and Tables

**Table 1:** Arithmetic mean and standard deviation of obstacle-crossing parameters for each trial and for their mean.

Condition	Parameter	T1	T2	T3	T123
		Mean (SD)	Mean (SD)	Mean (SD)	Mean (SD)
Approaching phase	Stride double support time (s)	0.36 (0.05)	0.35 (0.06)	0.35 (0.05)	0.36 (0.05)
	Stride duration (s)	1.10 (0.13)	1.09 (0.11)	1.09 (0.10)	1.09 (0.11)
	Stride length (cm)	131 (11.4)	131 (11.5)	132 (11.2)	131 (11.3)
	Stride velocity (cm/s)	120 (17.2)	122 (15.5)	122 (15.4)	121 (15.9)
	Stride width (cm)	10.6 (2.49)	11.3 (2.74)	11.2 (2.86)	11.0 (2.70)
Crossing phase	Step double support time (s)	0.22 (0.04)	0.20 (0.05)	0.21 (0.04)	0.21 (0.04)
	Step duration (s)	0.64 (0.08)	0.62 (0.07)	0.63 (0.07)	0.63 (0.07)
	Step length (cm)	72.7 (9.49)	73.4 (9.78)	73.6 (9.33)	73.2 (9.47)
	Step velocity (cm/s)	115 (20.8)	119 (21.5)	118 (19.0)	118 (20.4)
	Step width (cm)	10.2 (3.40)	10.4 (3.80)	10.1 (3.79)	10.2 (3.64)
Obstacle clearance	Leading foot horizontal distance prior to obstacle (cm)	108 (13.0)	108 (10.4)	108 (10.1)	108 (11.1)
	Trailing foot horizontal distance prior to obstacle (cm)	43.0 (10.3)	44.1 (8.77)	42.9 (10.1)	43.3 (9.71)
	Leading foot horizontal distance after obstacle (cm)	25.7 (6.61)	25.0 (6.63)	26.8 (7.75)	25.8 (7.00)
	Trailing foot horizontal distance after obstacle (cm)	91.6 (11.5)	91.8 (10.4)	95.0 (12.1)	92.8 (11.4)
	Leading foot toe clearance (cm)	9.93 (4.21)	9.99 (4.37)	10.8 (5.19)	10.2 (4.59)
	Trailing foot toe clearance (cm)	31.4 (8.68)	32.2 (8.01)	32.0 (6.53)	31.9 (7.74)

Note: SD: arithmetic standard deviation, T1: trial 1, T2: trial 2, T3: trial 3, T123: arithmetic mean of the three trials.

**Table 2:** Relative and absolute reliability for each obstacle-crossing parameter between the mean of the first and second trials (T1–T2), the first and third trials (T1–T3), the second and third trials (T2–T3), and the means of the three consecutive trials (T1–T2–T3).

Condition	Parameter	T1–T2					T1–T3					T2–T3					T1–T2–T3				
		ICC(2.1) [95%CI]	MDC	MDC%	SEM	SEM%	ICC [95%CI]	MDC	MDC%	SEM	SEM%	ICC(2.1) [95%CI]	MDC	MDC%	SEM	SEM%	ICC(2.1) [95%CI]	MDC	MDC%	SEM	SEM%
Approaching phase	Stride length (cm)	0.75 [0.58; 0.85]	15.9	12.2	5.75	4.40	0.76 [0.60; 0.86]	15.2	11.6	5.48	4.18	0.86 [0.76; 0.92]	11.7	8.94	4.23	3.23	0.79 [0.68; 0.87]	14.4	11.0	5.19	3.96
	Stride width (cm)	0.74 [0.55; 0.85]	3.71	34.0	1.34	12.3	0.74 [0.56; 0.85]	3.81	35.0	1.37	12.6	0.76 [0.61; 0.87]	3.75	33.4	1.35	12.1	0.75 [0.62; 0.84]	3.76	34.2	1.35	12.3
	Stride double support time (s)	0.76 [0.60; 0.87]	0.07	20.4	0.03	7.36	0.77 [0.61; 0.87]	0.07	18.8	0.02	6.78	0.81 [0.68; 0.89]	0.06	18.3	0.02	6.60	0.78 [0.67; 0.87]	0.07	19.2	0.02	6.92
	Stride duration (s)	0.87 [0.77; 0.93]	0.12	10.9	0.04	3.95	0.84 [0.72; 0.91]	0.13	11.5	0.05	4.15	0.87 [0.78; 0.93]	0.11	9.73	0.04	3.51	0.86 [0.78; 0.92]	0.12	10.7	0.04	3.88
	Stride velocity (cm/s)	0.83 [0.70; 0.90]	18.9	15.6	6.82	5.64	0.82 [0.69; 0.90]	19.2	15.8	6.92	5.71	0.90 [0.82; 0.95]	13.4	11.0	4.85	3.98	0.85 [0.76; 0.91]	17.3	14.3	6.26	5.16
Crossing phase	Step length (cm)	0.84 [0.72; 0.91]	10.8	14.8	3.89	5.33	0.81 [0.68; 0.89]	11.3	15.4	4.07	5.57	0.87 [0.77; 0.93]	9.52	13.0	3.44	4.67	0.84 [0.75; 0.90]	10.5	14.4	3.80	5.19
	Step width (cm)	0.35 [0.06; 0.59]	8.02	77.9	2.89	28.1	0.32 [0.02; 0.56]	8.20	80.8	2.96	29.2	0.71 [0.52; 0.83]	5.64	54.8	2.03	19.8	0.47 [0.28; 0.64]	7.37	72.0	2.66	26.0
	Step double support time (s)	0.19 [−0.10; 0.45]	0.11	53.5	0.04	19.3	0.44 [0.18; 0.65]	0.09	40.9	0.03	14.8	0.65 [0.44; 0.79]	0.07	34.4	0.03	12.4	0.42 [0.23; 0.60]	0.09	43.9	0.03	15.8
	Step duration (s)	0.60 [0.37; 0.76]	0.13	20.3	0.05	7.34	0.78 [0.63; 0.87]	0.09	14.9	0.03	5.38	0.76 [0.61; 0.86]	0.09	14.8	0.03	5.34	0.71 [0.58; 0.82]	0.11	16.9	0.04	6.09
	Step velocity (cm/s)	0.72 [0.54; 0.84]	31.0	26.5	11.2	9.54	0.77 [0.62; 0.87]	26.3	22.5	9.49	8.14	0.82 [0.69; 0.90]	24.0	20.2	8.65	7.29	0.77 [0.65; 0.86]	27.3	23.2	9.83	8.36
Obstacle clearance	Trailing foot horizontal distance prior to obstacle (cm)	0.74 [0.58; 0.85]	13.4	30.8	4.83	11.1	0.70 [0.51; 0.82]	15.5	36.0	5.58	13.0	0.80 [0.67; 0.89]	11.6	26.6	4.18	9.60	0.75 [0.62; 0.84]	13.6	31.3	4.89	11.3
	Trailing foot horizontal distance after obstacle (cm)	0.72 [0.54; 0.84]	15.9	17.4	5.74	6.26	0.71 [0.51; 0.84]	17.6	18.9	6.35	6.81	0.63 [0.41; 0.79]	18.9	20.3	6.83	7.32	0.69 [0.54; 0.80]	17.6	18.9	6.33	6.82
	Trailing foot toe clearance (cm)	0.67 [0.47; 0.81]	13.2	41.4	4.76	15.0	0.58 [0.34; 0.75]	13.8	43.5	4.98	15.7	0.73 [0.55; 0.84]	10.5	32.7	3.79	11.8	0.66 [0.51; 0.78]	12.6	39.4	4.53	14.2
	Leading foot horizontal distance prior to obstacle (cm)	0.51 [0.25; 0.70]	22.6	21.0	8.17	7.57	0.51 [0.25; 0.70]	22.5	20.9	8.12	7.55	0.69 [0.49; 0.82]	15.8	14.6	5.70	5.28	0.56 [0.39; 0.71]	20.5	19.1	7.41	6.88
	Leading foot horizontal distance after obstacle (cm)	0.71 [0.53; 0.83]	9.80	38.6	3.54	13.9	0.61 [0.38; 0.76]	12.5	47.6	4.51	17.2	0.70 [0.50; 0.82]	11.0	42.6	3.98	15.4	0.67 [0.52; 0.79]	11.2	43.2	4.03	15.6
	Leading foot toe clearance (cm)	0.84 [0.73; 0.91]	4.72	47.4	1.70	17.1	0.78 [0.63; 0.88]	6.14	59.3	2.21	21.4	0.89 [0.79; 0.94]	4.47	43.0	1.61	15.5	0.84 [0.75; 0.90]	5.16	50.4	1.86	18.2

Note: ICC: intraclass correlation coefficient, MDC: minimal detectable change, SEM: standard error of measurement, T1: trial 1, T2: trial 2, T3: trial 3. Mean relative reliability legend: slight (gray: 0.00 < ICC(2.1) < 0.20), fair (red: 0.21 < ICC(2.1) < 0.40), moderate (orange: 0.41 < ICC(2.1) < 0.60), substantial (yellow: 0.61 < ICC(2.1) < 0.80), and almost perfect (green: 0.81 < ICC(2.1) < 1.00). MDC% legend: low (yellow: MDC % ≤ 20%), acceptable (orange: 20% < MDC% < 40%), and high (red: MDC% ≥ 40%). SEM% legend: low (green: SEM % ≤ 10%), and high (SEM% > 10%).

**Table 3:** Agreement between the mean of the first and second trials (T1–T2), the first and third trials (T1–T3), and the second and third trials (T2–T3), for each obstacle-crossing parameter.

Condition	Parameter	T1–T2				T1–T3				T2–T3			
		CCC [95%CI]	LOA [95%CI]	Shieh Test [95%CI]	SWC	CCC [95%CI]	LOA [95%CI]	Shieh Test [95%CI]	SWC	CCC [95%CI]	LOA [95%CI]	Shieh Test [95%CI]	SWC
Approaching phase	Stride length (cm)	0.74 [0.57; 0.85]	[−16.1; 16.3]	[−18.7; 18.9]	2.14	0.76 [0.60; 0.86]	[−16.3; 14.3]	[−18.8; 16.8]	2.12	0.86 [0.75; 0.92]	[−12.8; 10.6]	[−14.7; 12.5]	2.19
	Stride width (cm)	0.74 [0.57; 0.84]	[−4.23; 2.85]	[−4.80; 3.42]	0.49	0.73 [0.57; 0.84]	[−4.30; 3.09]	[−4.90; 3.68]	0.50	0.76 [0.60; 0.86]	[−3.73; 3.89]	[−4.34; 4.50]	0.53
	Stride double support time (s)	0.76 [0.60; 0.86]	[−0.06; 0.08]	[−0.07; 0.09]	0.01	0.77 [0.62; 0.87]	[−0.05; 0.08]	[−0.06; 0.09]	0.01	0.81 [0.68; 0.89]	[−0.07; 0.07]	[−0.08; 0.08]	0.01
	Stride duration (s)	0.87 [0.77; 0.92]	[−0.10; 0.13]	[−0.12; 0.15]	0.02	0.84 [0.73; 0.90]	[−0.11; 0.14]	[−0.13; 0.16]	0.02	0.87 [0.78; 0.93]	[−0.11; 0.11]	[−0.13; 0.12]	0.02
	Stride velocity (cm/s)	0.82 [0.70; 0.90]	[−20.5; 17.5]	[−23.6; 20.5]	3.13	0.82 [0.69; 0.89]	[−21.2; 16.9]	[−24.3; 19.9]	3.11	0.90 [0.82; 0.94]	[−14.3; 13.0]	[−16.4; 15.2]	3.01
Crossing phase	Step length (cm)	0.83 [0.71; 0.91]	[−11.6; 10.1]	[−13.4; 11.9]	1.85	0.81 [0.67; 0.89]	[−12.2; 10.4]	[−14.1; 12.2]	1.79	0.87 [0.77; 0.93]	[−9.83; 9.53]	[−11.4; 11.1]	1.85
	Step width (cm)	0.34 [0.05; 0.58]	[−8.39; 7.85]	[−9.70; 9.15]	0.59	0.31 [0.01; 0.55]	[−8.29; 8.34]	[−9.63; 9.68]	0.58	0.70 [0.52; 0.83]	[−5.40; 6.00]	[−6.32; 6.92]	0.70
	Step double support time (s)	0.19 [−0.10; 0.44]	[−0.09; 0.13]	[−0.11; 0.14]	0.01	0.44 [0.17; 0.64]	[−0.07; 0.10]	[−0.09; 0.11]	0.01	0.64 [0.43; 0.79]	[−0.08; 0.06]	[−0.09; 0.08]	0.01
	Step duration (s)	0.59 [0.37; 0.75]	[−0.11; 0.14]	[−0.13; 0.16]	0.01	0.77 [0.62; 0.87]	[−0.09; 0.10]	[−0.10; 0.12]	0.01	0.76 [0.60; 0.86]	[−0.10; 0.09]	[−0.12; 0.10]	0.01
	Step velocity (cm/s)	0.71 [0.53; 0.83]	[−34.6; 26.7]	[−39.5; 31.7]	3.93	0.77 [0.61; 0.87]	[−28.9; 23.7]	[−33.1; 27.9]	3.75	0.81 [0.68; 0.89]	[−22.9; 25.6]	[−26.8; 29.5]	3.86
Obstacle clearance	Trailing foot horizontal distance prior to obstacle (cm)	0.74 [0.57; 0.85]	[−14.5; 12.3]	[−16.7; 14.5]	1.79	0.69 [0.50; 0.82]	[−15.6; 15.8]	[−18.2; 18.4]	1.88	0.80 [0.67; 0.88]	[−10.3; 12.7]	[−12.2; 14.6]	1.80
	Trailing foot horizontal distance after obstacle (cm)	0.72 [0.54; 0.83]	[−16.3; 16.1]	[−18.9; 18.7]	2.03	0.71 [0.53; 0.83]	[−20.1; 13.4]	[−22.8; 16.1]	2.20	0.63 [0.42; 0.77]	[−21.5; 15.1]	[−24.5; 18.0]	2.05
	Trailing foot toe clearance (cm)	0.67 [0.46; 0.80]	[−14.1; 12.4]	[−16.3; 14.6]	1.53	0.57 [0.34; 0.73]	[−14.6; 13.3]	[−16.8; 15.6]	1.36	0.72 [0.55; 0.84]	[−10.4; 10.9]	[−12.2; 12.6]	1.36
	Leading foot horizontal distance prior to obstacle (cm)	0.50 [0.25; 0.69]	[−23.4; 22.5]	[−27.1; 26.2]	2.04	0.50 [0.25; 0.69]	[−22.7; 23.0]	[−26.4; 26.7]	2.01	0.68 [0.48; 0.81]	[−15.4; 16.6]	[−18.0; 19.2]	1.88
	Leading foot horizontal distance after obstacle (cm)	0.71 [0.52; 0.83]	[−9.14; 10.6]	[−10.7; 12.2]	1.22	0.60 [0.38; 0.76]	[−13.6; 11.5]	[−15.6; 13.5]	1.29	0.69 [0.51; 0.82]	[−12.5; 9.02]	[−14.2; 10.7]	1.33
	Leading foot toe clearance (cm)	0.84 [0.72; 0.91]	[−4.86; 4.73]	[−5.63; 5.51]	0.82	0.78 [0.64; 0.87]	[−6.88; 5.18]	[−7.85; 6.15]	0.89	0.88 [0.81; 0.93]	[−5.07; 3.50]	[−5.76; 4.19]	0.93

Note: CCC: Lin’s concordance correlation coefficient, LOA: limits of agreement, SWC: smallest worthwhile change, T1: trial 1, T2: trial 2, T3: trial 3. CCC legend: Poor (red: CCC ≤ 0.40), moderate (yellow: 0.40 < CCC < 0.70), and good (green: CCC ≥ 0.70) agreement.

## Data Availability

The datasets used and/or analysed during the current study are available from the corresponding author on reasonable request.

## Supplementary Materials

The following supporting information can be downloaded at: https://www.mdpi.com/article/10.3390/s24113387/s1. S1. List of R packages used for statistical analysis [82,83,84,85,86,87].
