# Supplementary material for: Reliability of Obstacle-Crossing Parameters during Overground Walking in Young Adults
Source: Sensors (Basel). 2024 May 24;24(11):3387. doi: 10.3390/s24113387 (PMC11174552; doi:10.3390/s24113387)
Supplement: Supplementary file 1 [file sensors-24-03387-s001.zip › sensors-2974225-supplementary.pdf]

Supplementary Material

S1: List of R packages used for statistical analysis

The tidyverse package version 2.0.0 (including ggplot2) was used to handle, analyze, and present data (tables and graphics) [82].

The rstatix package version 0.7.2 was used to conduct the RM-ANOVA and and the pairwise comparison post hoc test [83].

The psych packge version 2.4.3 was used to compute the ICC(2.1) [84].

The psr package version 0.1.0 was used to determine the MDC, SEM, and SWC [85].

The SimplyAgree package version 0.2.0 was used to calculate the LOA, CCC and performed the Shieh exact test [86].

The blandr package version 0.5.1 was used to generate the Bland–Altman plots and compute the LOA [87].

References

82 Wickham, H.; Averick, M.; Bryan, J.; Chang, W.; D', L.; Mcgowan, A.; François, R.; Grolemund, G.; Hayes, A.; Henry, L.; et al. Welcome to the Tidyverse. J Open Source Softw **2019**, 4, 1686, doi:10.21105/JOSS.01686.

83 Kassambara, A. Pipe-Friendly Framework for Basic Statistical Tests [R Package Rstatix Version 0.7.2]. **2023**.

84 Revelle, W. Procedures for Psychological, Psychometric, and Personality Research [R Package Psych Version 2.4.3]. **2024**.

85 Huebner, A.; McGinn, T.; Sisk, M. psr: Functions for Analyzing Performance Science Data. **2021**

86 Caldwell, A. SimplyAgree: An R Package and Jamovi Module for Simplifying Agreement and Reliability Analyses. J Open Source Softw **2022**, 7, 4148, doi:10.21105/joss.04148.

87 Datta, D. blandr: a Bland-Altman Method Comparison package for R. Zenodo. **2017**
